# The Untold Story of Community Mobilizers Re-engaging a Disengaged Community During the Endemic Era of India's Polio Eradication Program

**DOI:** 10.9745/GHSP-D-20-00425

**Published:** 2021-03-15

**Authors:** Roma Solomon

**Affiliations:** a CORE Group Polio Project, Gurgaon, India.

## Abstract

Although India's polio eradication program began with a flourish in 1995, gradually, the community disengaged from the program as misinformation about the vaccine spread. Vaccination teams faced abuse and even physical aggression. What caused this break in communication? CORE Group Polio Project's mobilizers had to delve deep to uncover untold stories of why communities were disengaged from the government's polio eradication efforts.

## Key Messages

- Programs, no matter how vital, cannot be pushed into communities without proper information preceding them.
- People are suspicious of handouts especially when there is no rapport with the service providers. Misinformation will lead to suspicion and refusals.
- Good public health infrastructure engenders trust between the health service providers and communities.
- Policy makers need to realize that even the most disenfranchised have entitlements.
- Communication skills among the frontline workers are a must.
- Program managers would do well to interact with the decision makers in the community and take their inputs before planning any intervention.

## BACKGROUND

In 1995, the India polio eradication program began in earnest as the Pulse Polio Initiative, targeting all children under 5 years old. The program dispensed the oral polio vaccine to children nationwide through campaigns at kiosks set up at fixed sites twice a year. The vaccine was badly needed because in the mid-1990s, an estimated 150,000 polio cases were reported annually in India.^1^^,^^2^ Starting with a well-advertised flourish, the campaign drew crowds. Because polio was a dreaded and visible disease, people were eager to get their children protected and came willingly.

However, it soon became apparent that many children were being missed in these national immunization campaigns.^3^ In 1999, the government decided to send frontline health workers (FLWs), such as auxiliary nurse-midwives and other government-trained workers, as vaccinators to people's houses. The door-to-door campaign triggered larger scale suspicion because of the government's previous family planning efforts at the cost of other public health and sanitation improvements. Local suspicion was fueled by the fact that children were still becoming paralyzed by polio after being vaccinated.^2^ In my personal conversations with community members, I realized that people seemed to be suspicious of government intentions when the polio vaccine was made available at their doorsteps because no other vaccine was so conveniently, freely, and repeatedly provided.

In states like Uttar Pradesh, vaccinators were met with refusals, sometimes accompanied by abuses and physical aggression as experienced by our own teams. By rejecting the vaccine, people also got a chance to vent their long-standing grievances against inefficient health services. First, the auxiliary nurse-midwives and later, the accredited social health activists, came under fire and bore the brunt of the refusals.

The much-touted “People's Program” descended into what began to be perceived as a “Government Pogrom” corroborated by house markings left by the vaccinator—viewed as sinister symbols identifying certain populations. Was a certain community being targeted with a different vaccine? Did 2 drops of vaccine mean they could only have 2 children? This break in communication led to community disengagement.

Was it merely a refusal by people to accept the polio vaccine or was it something else?

The annals of war are usually written by generals and strategists, and many stories from the trenches remain untold. However, the soldiers return with tales of acts of valor, defeat, and victory—all contributing to not only the outcome of the war but, more importantly, a change in their personas. This is the story of a band of ordinary people who were hired to promote the polio vaccine—a seemingly innocuous task that turned into a war. How the war was won needs to be told so that the lessons can be used for other community interventions.

How the war against polio in India was won needs to be told so that lessons can be used for other community health interventions.

## CORE GROUP POLIO PROJECT EFFORTS IN INDIA

In 2003, CORE Group Polio Project* placed community mobilization coordinators (CMCs)^1^ at the frontline of this battle in Uttar Pradesh, the most populous and politicized Indian state and one of the first to become overtly hostile toward the polio program. The CMCs' job was to support the FLW vaccinators by mobilizing families to accept the polio vaccine—a seemingly easy task because they were selected locally. All vaccines were given at both institutional and outreach sessions at predetermined sites, and parents would not need so much motivation to bring their children.

## FACTORS CONTRIBUTING TO VACCINE REFUSAL

The community's refusals of the vaccine, therefore, were most unexpected since the families knew the CMCs. As it turned out, the polio vaccine became the target of people's anger, which stemmed from factors like substandard health service delivery leading to more out-of-pocket expenses, which in turn, affected their lives.^4^ Based on conversations with individuals in southern states in India, because good public health infrastructure had engendered trust between the health service providers and communities, the vaccine was not rejected because services had been provided to them as an entitlement and not as a handout. Polio was eliminated there sooner than in northern states like Uttar Pradesh and Bihar,^5^ where people had to spend money on private health care, leading to debt and poverty.^5^ A repeated, coercive, and “doorstep” vaccine campaign lit the spark that would trigger large-scale refusals.

The vaccine wasn't rejected in southern India states because services had been provided to them as an entitlement, not as a handout.

Accompanying the FLW vaccinators in Varanasi, Uttar Pradesh, one day, we came across a weaver sitting at his doorstep, blocking our entry. Holding a beautifully woven piece of silk in his hands, he was crying with frustration and anger. He told us that the import of cheap Chinese artificial silk had flooded the market, killing the weaving industry to such an extent that he could not even buy food. Government policies like these would destroy the centuries-old craft that Varanasi was famous for, and impoverish its artisans. The weaver's priority was food, not the polio vaccine. Barring India's coercive family planning program of the 1970s, no other government initiative had evoked so much anger in recent times. To avoid another repeat reaction, the people needed to be heard, no matter how trivial their grievances may have appeared. It was also vital that communities received correct information about the polio vaccine and not assume that they would accept whatever was being offered.

Sometimes, the cause behind a refusal can be avoided. A CMC led us to a house where the husband had strictly forbidden any FLW vaccinator to enter, thus it had no door markings. When the wife opened the door and saw the CMC, she started shouting at us and told us to leave. Upon asking, she blurted out the reason behind her behavior. A couple of months ago, her son had developed paralysis of one leg. Because this was a symptom of polio, the surveillance officer took a stool sample from her son and left. The family was boycotted by the community because they feared that the son had polio. In fact, the son did not have polio. The parents demand for a letter or certificate clarifying that—a simple demand and logical step that could have been easily addressed—was never met. Not having the letter, in turn, barred all vaccinator visits, and all the children remained unimmunized.

There were many such instances where parents needed reassurance through reasoning that the vaccine would not harm their child. However, this effort required time and skill.

The CMCs were placed in the harshest places where the population was the poorest, most disenfranchised, neglected, unreached, migratory, and not registered in government records. To begin with, they were not trained communicators, just young girls, literate enough to collect and record data, most having never worked before. At the first residential training, some came accompanied by a sibling or parent. It had become clear that for them to succeed, they had to have people's acceptance, therefore, their training concentrated on interpersonal communication skills, coupled with basic technical knowledge about vaccination and polio. They learned to talk to parents and seniors like mothers in-law and delve deep into their minds to understand where the negative behavior was coming from.

A very important component of their work was to support and accompany the FLW vaccinators from house to house, and this required a lot of mutual adjustment. The FLWs were older, experienced, and technically qualified, whereas the CMCs were younger, fresh out of school, or at home. However, both parties soon realized that close cooperation was the only means of succeeding. Their roles became clearer: the CMCs prepped the parents for the vaccine, and the FLWs delivered it. Thus, the CMCs earned a valid place for themselves in the program and became a vital resource for reaching people.

CMCs—tasked with supporting FLWs in helping families accept the vaccine—realized that they had to closely cooperate with FLWs to succeed.

It soon became evident to the CMCs that making significant progress into homes would not be possible without the help of “influencers” who had a standing in the community such as village and religious leaders, local doctors, etc. These individuals agreed to accompany the vaccinators to address refusals and gave their time voluntarily. They held regular community meetings to answer questions, especially those pertaining to religious beliefs that were some of the hardest to address.

The FLW vaccinators, being government appointed, were not accustomed to refusals. However, their air of authority quickly disappeared in the face of sullen or even aggressive reactions. The CMCs had a different skill set, more empathetic body language, and simple tools that calmed angry parents and explained why the vaccine was important for their children. The CMCs let the vaccinators do their job and promised to return to not only check on the child but also discuss other health issues since their training skill set now also included messages on water and sanitation, diarrhea management, antenatal care, breastfeeding, etc. These repeated visits to track children's health and immunization started bringing down the barriers.

Mothers' groups were formed to discuss health issues. Fathers were accessed through other contacts like local barbers who were trained to initiate conversations about immunization in general and polio while they cut hair. Schoolchildren carried messages on the importance of hygiene to their homes.

All of these efforts helped to build bridges between the people and the program. Slowly, the polio eradication program began to be accepted and owned by those for whom it was meant. More importantly, the government functionaries and CMCs worked as a team, sharing their maps, material, and data with each other.

## CONCLUSION

In summary, community engagement needs to be on the agenda of any public health program from the start and not viewed as a separate objective. In fact, it is the most valued indicator of success.

The tide of acceptance of the polio vaccine turned with the realization that the most important people were actually those for whom the program was intended. This significant shift occurred when the people who were trying to change the community's behavior realized that they themselves also had to undergo transformation in their own attitudes.

The CMCs had to pass on the skills that they had acquired through experience and practice to the FLWs, especially to the accredited social health activists who were closest to the communities. FLWs, whether from the government or elsewhere, had to change by perceiving the “beneficiaries” as “clients”—the latter designation garnering more respect. The FLWs also had to listen and respond to people's other health complaints. This responsive climate became the new normal, building trust between the community and program staff until India was officially declared polio-free on March 27, 2014.

## Footnotes

* An ongoing U.S. Agency for International Development-funded initiative started in 1999 in India and other polio-endemic countries to assist governments in community mobilization and surveillance for polio through civil society organization consortia.

## Notes

**Peer Reviewed**
